# Supplementary material for: Modeling hepatitis C virus kinetics during liver transplantation reveals the role of the liver in virus clearance
Source: eLife. 2021 Nov 3;10:e65297. doi: 10.7554/eLife.65297 (PMC8608386; doi:10.7554/eLife.65297)
Supplement: Supplementary file 1. — Best-fit parameter estimates determined by fitting Equation. (3) with data obtained during the anhepatic (AH) phase, assuming extracellular fluid volume of 5 L and that fluid intake and outtake are equal (see Materials and methods). VP, viral plateau (not significantly different from slope 0). Note that the median is not provided because many patients had a best-fit value of 0 for the slope precluding an estimate of a half-life. [file elife-65297-supp1.docx]

| **Phase** | **Case** | **HCV t_1/2_ [min]**  **[95% CI]** |
| --- | --- | --- |
| AH | 1 | VP |
|  | 2 | VP |
|  | 3 | VP |
|  | 4 | 64 [61-69] |
|  | 5 | 89 [75-110] |

**Table S1:** Best-fit parameter estimates determined by fitting Eqs. (3) with data obtained during the anhepatic phase (AH), assuming extracellular fluid volume of 5L and that fluid intake and outtake are equal (see Methods). VP, viral plateau (not significantly different from slope 0). Note that the median is not provided because many patients had a best-fit value of 0 for the slope precluding an estimate of a half-life
