# Supplementary material for: Modeling hepatitis C virus kinetics during liver transplantation reveals the role of the liver in virus clearance
Source: eLife. 2021 Nov 3;10:e65297. doi: 10.7554/eLife.65297 (PMC8608386; doi:10.7554/eLife.65297)
Supplement: Supplementary file 2. — Best-fit parameter estimates determined by fitting Equation. (4) with data obtained during the 4 hr after graft reperfusion (RP), assuming extracellular fluid volume of 5 L and that fluid intake and outtake are equal (see Materials and methods). VP, viral plateau (not significantly different from slope 0). * Since c0 and cRP were highly correlated and not independently identifiable (correlation matrix) and population modeling (using Monolix) indicated that c0 was not identifiable (not shown), the initial virus clearance rate (i.e., c0 in Equation. 4) was fixed to its best-fit value (first estimated with c0, cRP, and κ as free parameters) and then the errors on the remaining parameters (cRP and κ) were computed. ** Equation. 3 was used to estimate hepatitis C virus (HCV) t1/2. Since best estimate of clearance was cRP = 0, half-life is undefined. [file elife-65297-supp2.docx]

| **Phase** | **Case** | **Init. HCV t_1/2_* [min]** | **κ**  **[1/days]**  **[95% CI]** | **HCV t_1/2_ [min]**  **[95% CI]** |
| --- | --- | --- | --- | --- |
|  | 1 | 2.9 | 143 [120-166] | 77 [53-140] |
| RP | 2 | 0.3 | 5000 [4600- 5400] | 57 [53-62] |
|  | 3 | 1.9 | 399 [348, 450] | 80 [65-107] |
|  | 4 | 16.9 | 100^†^ | 78 [66-96] |
|  | 5 | - | - | VP** |
|  | **Median (range)** | **2.4**  **(0.3-16.9)** | **399**  **(143-5000)** | **67**  **(57-80)** |

**Table S2:** Best-fit parameter estimates determined by fitting Eqs. (4) with data obtained during the 4h after graft reperfusion (RP), assuming extracellular fluid volume of 5L and that fluid intake and outtake are equal (see Methods). VP, viral plateau (not significantly different from slope 0).

* Since *c_0_* and *c_rp_* were highly correlated and not independently identifiable (correlation matrix) and population modeling (using Monolix) indicated that c0 was not identifiable (not shown), the initial virus clearance rate (i.e., c_0_ in Eq. 4) was fixed to its best-fit value (first estimated with *c_0_*, *c_rp_*, and κ as free parameters) and then the errors on the remaining parameters (*c_rp_*, and κ) were computed.

** Eq. 3 was used to estimate HCV t_1/2_. Since best estimate of clearance was *c_RP_=0*, half-life is undefined.
