# Supplementary material for: Modeling hepatitis C virus kinetics during liver transplantation reveals the role of the liver in virus clearance
Source: eLife. 2021 Nov 3;10:e65297. doi: 10.7554/eLife.65297 (PMC8608386; doi:10.7554/eLife.65297)
Supplement: Supplementary file 3. — Longer term viral kinetics. We found the time when hepatitis C virus (HCV) VL begins increasing and then determined the half-life of HCV up until the time of the first increase. We then measured the continued decrease from the end of reperfusion (RP) until this time and determined the slope using linear regression. We converted this slope to a half-life and found that the half-life tends to be on the order of several hours (except for Case 5 in whom a viral plateau was observed until the end of the follow-up period). [file elife-65297-supp3.docx]

| **Case** | **Time between 4h post-RP until HCV rebound [h]** | **HCV t_1/2_ until rebound [h]** |
| --- | --- | --- |
| 1 | 9.7 | 3.3 |
| 2 | 5.1 | 1.7 |
| 3 | 15.5 | 4.8 |
| 4 | 10.6 | 4.2 |
| 5* | - | - |
| **Median (range)** | **10.2**  **(5.1-15.5)** | **4.2**  **(1.7-70.6)** |

*Note that for Case 5 there was no resurgence.

**Table S3:** Longer term viral kinetics. We found the time when HCV VL begins increasing and then determined the half-life of HCV up until the time of the first increase. We then measured the continued decrease from the end of RP until this time and determined the slope using linear regression. We converted this slope to a half-life and found that the half-life tends to be on the order of several hours (except for Case 5 in whom a viral plateau was observed until the end of the follow up period).
