# Supplementary material for: Modeling hepatitis C virus kinetics during liver transplantation reveals the role of the liver in virus clearance
Source: eLife. 2021 Nov 3;10:e65297. doi: 10.7554/eLife.65297 (PMC8608386; doi:10.7554/eLife.65297)
Supplement: Transparent reporting form [file elife-65297-transrepform1.docx]

***eLife’s* transparent reporting form**

We encourage authors to provide detailed information *within their submission* to facilitate the interpretation and replication of experiments. Authors can upload supporting documentation to indicate the use of appropriate reporting guidelines for health-related research (see [EQUATOR Network](http://www.equator-network.org/%20)), life science research (see the [BioSharing Information Resource](https://biosharing.org/)), or the [ARRIVE guidelines](http://www.plosbiology.org/article/info:doi/10.1371/journal.pbio.1000412) for reporting work involving animal research. Where applicable, authors should refer to any relevant reporting standards documents in this form.

If you have any questions, please consult our Journal Policies and/or contact us: [editorial@elifesciences.org](mailto:editorial@elifesciences.org).

**Sample-size estimation**

- You should state whether an appropriate sample size was computed when the study was being designed
- You should state the statistical method of sample size computation and any required assumptions
- If no explicit power analysis was used, you should describe how you decided what sample (replicate) size (number) to use

Please outline where this information can be found within the submission (e.g., sections or figure legends), or explain why this information doesn’t apply to your submission:

This was an exploratory study in which we did not compare clinical/virological events, therefore, we did not include a simple size calculation. Rather the goal was to describe with maximun detail, HCV kinetics within individual patients during the two most important phases of surgery in liver transplantation: the anhepatic phase and the reperfusion phase. The strength of the study is in the very frequent kinetic data obtained.

**Replicates**

- 1) You should report how often each experiment was performed
- 2) You should include a definition of biological versus technical replication
- 3) The data obtained should be provided and sufficient information should be provided to indicate the number of independent biological and/or technical replicates
- 4) If you encountered any outliers, you should describe how these were handled
- 5) Criteria for exclusion/inclusion of data should be clearly stated
- 6) High-throughput sequence data should be uploaded before submission, with a private link for reviewers provided (these are available from both GEO and ArrayExpress)

Please outline where this information can be found within the submission (e.g., sections or figure legends), or explain why this information doesn’t apply to your submission:

1-3 – information found in M&M and figure legend

4 – No outlier data was left out.

5 – There were two samples in the 7 experiments reported in which the qPCR CTs were above 30 for both HCV and GAPDH indicating the RNA had likely been lost during isolation. These two samples were no included in the analysis (They were one sample of multiple replicates).

6 – not applicable

**Statistical reporting**

- Statistical analysis methods should be described and justified
- Raw data should be presented in figures whenever informative to do so (typically when N per group is less than 10)
- For each experiment, you should identify the statistical tests used, exact values of N, definitions of center, methods of multiple test correction, and dispersion and precision measures (e.g., mean, median, SD, SEM, confidence intervals; and, for the major substantive results, a measure of effect size (e.g., Pearson's r, Cohen's d)
- Report exact p-values wherever possible alongside the summary statistics and 95% confidence intervals. These should be reported for all key questions and not only when the p-value is less than 0.05.

Please outline where this information can be found within the submission (e.g., sections or figure legends), or explain why this information doesn’t apply to your submission:

## Statistical analysis method is described in Methods. Exact p-values are shown in Table 2.

(For large datasets, or papers with a very large number of statistical tests, you may upload a single table file with tests, Ns, etc., with reference to sections in the manuscript.)

**Group allocation**

- Indicate how samples were allocated into experimental groups (in the case of clinical studies, please specify allocation to treatment method); if randomization was used, please also state if restricted randomization was applied
- Indicate if masking was used during group allocation, data collection and/or data analysis

Please outline where this information can be found within the submission (e.g., sections or figure legends), or explain why this information doesn’t apply to your submission:

Not applicable

**Additional data files (“source data”)**

- We encourage you to upload relevant additional data files, such as numerical data that are represented as a graph in a figure, or as a summary table
- Where provided, these should be in the most useful format, and they can be uploaded as “Source data” files linked to a main figure or table
- Include model definition files including the full list of parameters used
- Include code used for data analysis (e.g., R, MatLab)
- Avoid stating that data files are “available upon request”

Please indicate the figures or tables for which source data files have been provided:

Source clinical data are shown Tables 1 and 4 and viral kinetics are shown in Figs. 1 and 3. In vitro data is shown in Fig. 4. We have provided source data files for Figures 3 and 4. Models codes are not provided since the mathematical models are simple ordinary differential equations. Code used to calibrate models with data are mentioned in Methods.
